# Timing of paediatric orchidopexy in universal healthcare systems: international administrative data cohort study

**DOI:** 10.1002/bjs5.50329

**Published:** 2020-07-24

**Authors:** M. A. Jay, A. Arat, L. Wijlaars, O. Ajetunmobi, T. Fitzpatrick, H. Lu, S. Lei, C. Skerritt, S. Goldfeld, M. Gissler, G. Gunnlaugsson, S. Hrafn Jónsson, A. Hjern, A. Guttmann, R. Gilbert

**Affiliations:** ^1^ Population Policy and Practice Research and Teaching Department University College London Great Ormond Street Institute of Child Health London UK; ^2^ Bristol Royal Hospital for Children Bristol UK; ^3^ Clinical Epidemiology, Department of Medicine, Karolinska Institutet and Centre for Health Equity Studies Stockholm Sweden; ^4^ Department of Neurobiology, Care Sciences and Society Karolinska Institutet Huddinge Sweden; ^5^ Child Health Evaluative Sciences Canada; ^6^ Division of Paediatric Medicine, Hospital for Sick Children Canada; ^7^ ICES Canada; ^8^ Dalla Lana School of Public Health Canada; ^9^ Department of Paediatrics University of Toronto Toronto Ontario Canada; ^10^ Murdoch Children's Research Institute, The Royal Children's Hospital Australia; ^11^ Department of Paediatrics University of Melbourne Parkville Victoria Australia; ^12^ Information Services Department Finnish Institute for Health and Welfare Helsinki Finland; ^13^ Faculty of Sociology, Anthropology and Folkloristics University of Iceland Reykjavík Iceland

## Abstract

**Background:**

International guidelines in 2008 recommended orchidopexy for undescended testis at 6–12 months of age to reduce the risk of testicular cancer and infertility. Using administrative data from England, Finland, Ontario (Canada), Scotland and Sweden (with data from Victoria (Australia) and Iceland in supplementary analyses), the aim of this study was to investigate compliance with these guidelines and identify potential socioeconomic inequities in the timing of surgery before 1 and 3 years.

**Methods:**

All boys born in 2003–2011 with a diagnosis code of undescended testis and procedure codes indicating orchidopexy before their fifth birthday were identified from administrative health records. Trends in the proportion of orchidopexies performed before 1 and 3 years of age were investigated, as were socioeconomic inequities in adherence to the guidelines.

**Results:**

Across all jurisdictions, the proportion of orchidopexies occurring before the first birthday increased over the study period. By 2011, from 7·6 per cent (Sweden) to 27·9 per cent (Scotland) of boys had undergone orchidopexy by their first birthday and 71·5 per cent (Sweden) to 90·4 per cent (Scotland) by 3 years of age. There was limited evidence of socioeconomic inequities for orchidopexy before the introduction of guidelines (2008). Across all jurisdictions for boys born after 2008, there was consistent evidence of inequities in orchidopexy by the first birthday, favouring higher socioeconomic position. Absolute differences in these proportions between the highest and lowest socioeconomic groups ranged from 2·5 to 5·9 per cent across jurisdictions.

**Conclusion:**

Consistent lack of adherence to the guidelines across jurisdictions questions whether the guidelines are appropriate.

## Graphical abstract

International guidelines recommend orchidopexy before 12 months of age. This study, using international administrative health data, found that this was achieved in only a minority of cases and that there were socioeconomic inequities in this outcome.

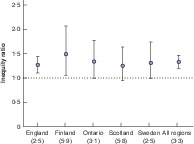

Guidelines not widely applied

## Abstract (es)

**Antecedentes:**

En el 2008, las guías internacionales recomendaban efectuar una orquidopexia para los testículos no descendidos entre los seis y los 12 meses de edad para reducir los riesgos de cáncer testicular e infertilidad. Utilizando datos administrativos de Inglaterra, Finlandia, Ontario (Canadá), Escocia y Suecia (con datos de Victoria, Australia e Islandia para análisis complementarios), el objetivo de este estudio fue investigar el cumplimiento de estas guías y la identificación de posibles desigualdades socioeconómicas con relación al momento de la cirugía antes de 1 y 3 años de edad.

**Métodos:**

A partir de los registros administrativos de salud, se identificaron todos los niños nacidos entre 2003 y 2011 con código diagnóstico de testículos no descendidos y con código de procedimiento correspondiente a orquidopexia antes de cumplir 5 años. Se investigaron las tendencias en la proporción de orquidopexias realizadas antes de 1 y 3 años de edad, respectivamente, al igual que las desigualdades socioeconómicas en el cumplimiento de las directrices de las guías.

**Resultados:**

En todas las jurisdicciones, la proporción de orquidopexias realizadas antes del primer año de vida aumentó durante el periodo de estudio. En 2011, del 7,6% (Suecia) al 27,9% (Escocia) de los niños habían sido sometidos a orquidopexia en su primer año de vida y del 71,5% (Suecia) al 90,4% (Escocia) a los 3 años de edad. Hubo evidencia limitada de las inequidades socioeconómicas para la orquidopexia antes de la introducción de las guías (2008). En todas las jurisdicciones para los niños nacidos después de 2008, hubo evidencia consistente de inequidades para la práctica de una orquidopexia en el primer año de vida en favor de una posición socioeconómica más alta (*socioeconomic position*, SEP). Las diferencias absolutas en estas proporciones entre los grupos SEP más altos y más bajos oscilaron entre el 2,5% y el 5,9% en todas las jurisdicciones.

**Conclusión:**

La falta de adherencia a las guías observada consistentemente en todas las jurisdicciones cuestiona si las guías son apropiadas.

## Introduction

Undescended testis is common, affecting around 3–9 per cent of full‐term boys at birth^1^. Most undescended testes spontaneously descend, but the problem persists beyond 1 year of age in around 1 per cent of individuals^1^. When uncorrected, undescended testis is associated with an increased risk of testicular cancer in adulthood as well as reduced testicular volume, sperm count and hormonal levels, with potential risks for fertility that may impact on psychosexual well‐being^2^, ^3^, ^4^, ^5^, ^6^, ^7^, ^8^, ^9^, ^10^, ^11^. Early surgical placement of the testis into the scrotum is recommended to reduce these risks.

Two European consensus statements^2^, ^4^ were published in 2008, followed by one from the British Association of Paediatric Urologists^12^ in 2011, recommending surgical intervention between 6 and 12 months of age. Although guidelines from the American Urological Association^13^ in 2014 recommended orchidopexy before the age of 18 months, a systematic review^14^ concluded that surgery between 6 and 12 months of age may optimize fertility and protect against malignancy. Older, single jurisdiction, studies have found poor adherence to these guidelines^15^, ^16^, ^17^, ^18^, ^19^, ^20^, ^21^, ^22^, ^23^.

Many healthcare systems provide free universal care. Equity of access is a key goal of these systems. A number of investigations have found, however, that people of lower socioeconomic position (SEP) are less likely to access healthcare services after adjusting for clinical need^24^, ^25^, ^26^, ^27^, ^28^. There are also concerns that public health interventions that do not address root causes of inequities in access to healthcare may lead to increased health inequity overall^29^. Orchidopexy is a useful exemplar condition to assess inequities in healthcare access, because the occurrence of undescended testis is not known to be associated with SEP^30^ and the condition is routinely screened for at birth.

The aims of this study were to investigate trends in the proportion of orchidopexies performed before 1 and 3 years of age in five jurisdictions (England, Finland, Ontario (Canada), Scotland and Sweden), and to investigate potential socioeconomic inequities in the proportion of boys from high‐ *versus* low‐SEP families having orchidopexy before 1 and 3 years of age, stratified according to the introduction of the guidelines in 2008.

## Methods

Rolling yearly male‐only birth cohorts were identified using administrative health data sets in five jurisdictions (England, Finland, Ontario, Scotland and Sweden) from 2003 to 2011. Owing to data limitations, the 2003 birth cohort was unavailable for Finland. All singleton live births among boys surviving to at least 6 months of age who had orchidopexy before their fifth birthday were included; the population denominator was the number of all singleton live‐born males in each respective year.

The data set for each jurisdiction was a whole‐population administrative data set from health services, from which it was possible to construct birth cohorts. Detail on each data set is available in *Table* *S1* (supporting information). Each data set contained information on demographic and clinical characteristics of the patients, as well as dates and procedure codes associated with their hospital admissions. Complementary data were also supplied for Iceland and Victoria (Australia), but were excluded from the main analyses because of data limitations. In Iceland, the number of boys undergoing orchidopexy in each year was very small; in Victoria, birth cohorts could not be constructed and cross‐sectional data only were provided. Direct comparisons with these jurisdictions were therefore not feasible. Results for these two regions are presented in the supporting information, as indicated in the results section.

### Case definition

A case was defined as any child born in 2003–2011 with hospital records indicating a diagnosis of undescended testis and who underwent orchidopexy by the age of 5 years. Orchidopexies were identified using jurisdiction‐specific procedure codes (*Table* *S1*, supporting information). As orchidopexy is also used to treat testicular torsion, patients were included only if they had an ICD‐10 cryptorchidism diagnosis code (*Table* *S1*, supporting information) recorded either on the same admission record as their orchidopexy or previously. Only the first operation was counted (revisions or second stages were ignored). Patients were followed up to their fifth birthday to reduce the risk of counting acquired ascending testis, which can occur during later childhood^31^.

Boys born preterm (at less than 37 weeks' gestation) were excluded, as were those with a congenital anomaly at birth, identified by any ICD‐10 diagnosis code indicating the presence of congenital anomalies in the first 3 months of life (*Appendix* *S1*, supporting information)^32^.

### Outcomes

Trends in the proportion of cases by age 5 years, where the first orchidopexy procedure was performed before 1 and 3 years of age, were plotted. Inequity ratios were then calculated for boys born before (2003–2006) and after (2008–2011) the introduction of European guidance recommending orchidopexy before 1 year of age. Inequity ratios were based on the distribution of births into quintiles (or quartiles for Finland) according to SEP using either household‐ or area‐level measures recorded in the birth or subsequent admission records.

### Ethical approval

Access to data was granted following appropriate approvals in each jurisdiction. In England, researchers had a data‐sharing agreement with National Health Service Digital to use a deidentified extract of Hospital Episode Statistics linked to Office for National Statistics death registration data, so that ethical approval to use English data sets was not required. In Iceland, the study was approved by the Data Protection Authority and National Bioethics Committee (7 March 2017, VSN–17–044), the Directorate of Health (20 January 2017, 1701096/5.6.1) and Statistics Iceland (24 April 2017, 2017/01). No study permission was required in Finland, as only aggregated data were provided for the study group. The use of encoded Ontario data, accessed at ICES in this project, was authorized under section 45 of Ontario's Personal Health Information Protection Act, which does not require review by a Research Ethics Board. The Public Benefit and Privacy Panel for Health and Social Care (reference number 1516‐0405) and the Privacy Advisory Committee (number XRB13020) provided permission in Scotland. The Swedish part of this study was approved by the Regional Ethics Committee in Stockholm in January 2016 (dnr 2016/5:1), and in Victoria this was covered by RCH HREC 37164.

Small cell counts were suppressed in accordance with the requirements of each jurisdiction.

### Statistical analysis

Trends based on the annual cumulative incidence of orchidopexy by age 5 years in each jurisdiction were plotted over time, based on year of birth. Trends in the proportion of patients undergoing orchidopexy before 1 and 3 years of age in each region by year of birth were also plotted. These proportions are cumulative: the proportion having orchidopexy by 3 years of age includes those who had it by 1 year.

Inequity in age at orchidopexy was measured by taking the proportion of patients in the three highest (2 highest in the case of Finland) SEP groups over the proportion of patients in the two lowest SEP groups who received their first orchidopexy by 1 or 3 years of age. These ratios are termed inequity ratios. A ratio of 1 indicates equality between the patients with higher and lower SEP. Ratios above 1 indicate inequities in favour of patients with a higher SEP, and those below 1 indicate inequities in favour of patients with a lower SEP. Ninety‐five per cent confidence intervals were also calculated. To assess inequities before and after the publication of the guidelines, inequity ratios were calculated for boys born in 2003–2006 (2004–2006 in Finland), and again for those born in 2008–2011.

Following up boys to their fifth birthday may have introduced bias into the analyses as later operations may nonetheless be for primary undescended testis. Sensitivity analyses were therefore carried out by extending follow‐up to the tenth birthday. Data were available for these analyses only from England, Ontario, Scotland and Sweden.

Analyses were conducted in each region independently, based on a detailed specification of the cohort and codes required. The statistical software used in each region included R (R Foundation for Statistical Computing, Vienna, Austria) (England, Iceland, Victoria), Stata® (StataCorp, College Station, Texas, USA) (Scotland) and SAS® (SAS Institute, Cary, North Carolina, USA) (Finland, Ontario and Sweden). Aggregate results were entered into Microsoft Excel® (Microsoft, Redmond, Washington, USA) for further analysis.

## Results

Characteristics of boys born in 2011 in each jurisdiction are given in *Table* *1* (all years are in *Appendix* *S2*, supporting information). In 2011, 331 104 boys were born in England, 69 177 in Ontario, 54 400 in Sweden, 30 566 in Finland, and 28 099 in Scotland. Further descriptive data on the distribution of maternal age, birthweight and the numbers excluded due to death at less than 6 months of age are given in *Appendix* *S2* (supporting information).

**Table 1:** Characteristics of all male births in the latest birth year by region

	England	Finland	Ontario	Scotland	Sweden
**Latest birth year**	2011	2011	2011	2011	2011
**No. of male births**	331 104	30 566	69 177	28 099	54 400
**Socioeconomic position at birth**					
Most deprived	90 122 (27·9)	6027 (23·5)	19 145 (27·7)	7349 (26·2)	15 938 (29·4)
2nd	73 403 (22·7)	11 513 (45·0)	14 145 (20·4)	6003 (21·4)	10 300 (19·0)
3rd	59 757 (18·5)	5325 (20·8)	13 272 (19·2)	5407 (19·3)	8980 (16·6)
4th	51 690 (16·0)	2744 (10·7)	12 517 (18·1)	4949 (17·6)	10 269 (18·9)
Least deprived	48 548 (15·0)	–*	10 098 (14·6)	4379 (15·6)	8722 (16·1)
**Any congenital anomaly**	9543 (2·9)	1514 (5·0)	2118 (3·1)	743 (2·6)	1122 (2·1)
**Premature birth (< 37 weeks' gestation)**	17 210 (5·7)	1764 (5·8)	5755 (8·3)	1590 (5·7)	2657 (4·9)
**Congenital anomaly or prematurity**	24 988 (8·2)	2759 (9·1)	7213 (10·4)	2180 (7·8)	3634 (6·7)

Values in parentheses are percentages (calculated by excluding missing values for each variable); data for all years and missing values are shown in *Appendix* *S2* (supporting information), where data for Iceland and Victoria, Australia, can also be found.

* Finland's socioeconomic position categorization is on a four‐point scale only.

**Fig. 1:**
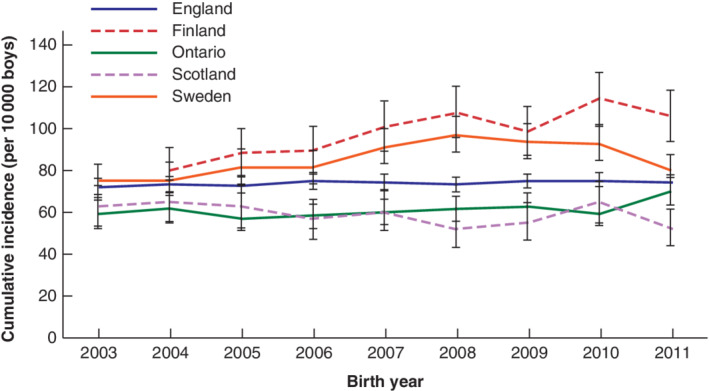
Cumulative incidence of orchidopexy by age 5 years in each region, 2003–2011 Error bars show 95 per cent confidence intervals.

*Fig*. *1* and *Appendix S3* (supporting information) show the cumulative incidence of cases over time in each region. Rates in England, Scotland and Ontario remained stable from 2003 to 2011. In Finland, the rate rose from 79·9 per 10 000 for boys born in 2004 to 105·6 for those born in 2011. Sweden observed an increased rate for boys born between 2006 and 2008, which then decreased from 2009 to 2011.

**Fig. 2:**
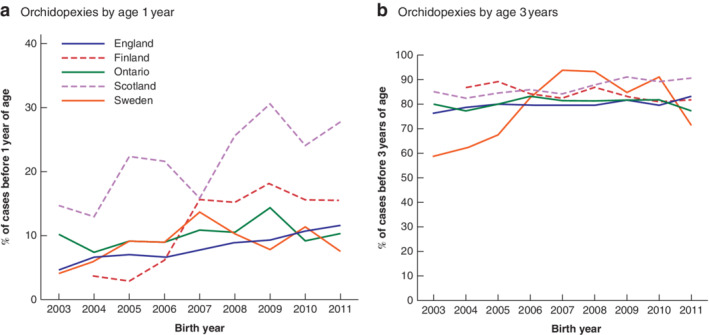
Percentage of orchidopexies performed by 1 and 3 years of age in each region, 2003–2011 Orchidopexies by **a** 1 year and **b** 3 years of age. Note the different scales on the *y*‐axes.

Across all jurisdictions, a minority of boys had orchidopexy before the recommended age of 1 year, but a large majority had undergone the procedure by 3 years (*Fig*. *2* and *Appendix S3*, supporting information). All regions, except Sweden, showed increases in the proportions of boys having orchidopexy by the age of 1 year, with the steepest rises in Scotland between 2007 and 2009 and Finland between 2006 and 2007. In Sweden, there was a rise in this proportion to 2007, which had then decreased by 2009 to 2004–2005 levels. The proportions receiving orchidopexy by age 3 years were more stable across all birth years, except for marked increases in Sweden from 2003 to 2007.

**Fig. 3:**
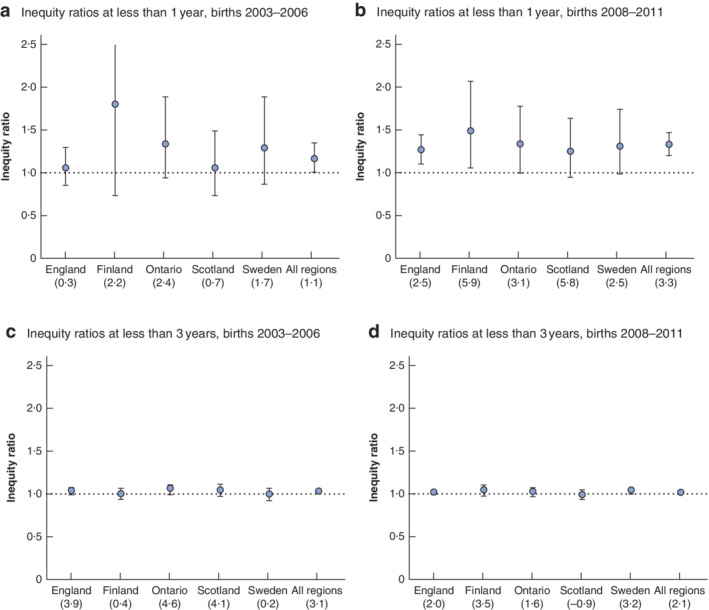
Inequity ratios for the timing of orchidopexy between the most deprived and least deprived patients, 2003–2006 and 2008–2011 **a,b** Inequity ratios at less than 1 year for births in **a** 2003–2006 (2004–2006 for Finland) and **b** 2008–2011. **c,d** Inequity ratios at less than 3 years for births in **c** 2003–2006 (2004–2006 for Finland) and **d** 2008–2011. Error bars show 95 per cent confidence intervals. The values in parentheses below each region show the absolute percentage difference (higher socioeconomic position (SEP) minus lower SEP).

*Fig*. *3* and *Appendix* *S4* (supporting information) give the inequity ratios in time to surgery in England, Finland, Ontario, Scotland and Sweden for the 1‐year threshold for birth years 2003–2006 (2004–2006 for Finland) (*Fig*. *3a*) and 2008–2011 (*Fig*. *3b*). The ratios for the 3‐year threshold are shown in *Fig*. *3c,d*. There was limited evidence of socioeconomic inequity in age at orchidopexy for births between 2003 and 2006, with small relative and absolute differences between boys with higher and lower SEP. For births between 2008 and 2011, however, there was consistent evidence across all jurisdictions of inequities for the first birthday guideline, favouring boys with a higher SEP. Boys born in 2008–2011 with a higher SEP were 25–48 per cent more likely to have had their operation by 1 year of age, depending on region, compared with those with a lower SEP. The absolute differences were small, and ranged from 2·5 to 5·9 per cent across each region.

There was limited evidence of very small inequities in the 3‐year threshold for births in 2003–2006 or 2008–2011.

Results of the sensitivity analyses are given in *Appendices S5* and *S6* (supporting information). There were no substantive differences between the main and sensitivity analyses in inequity ratios.

## Discussion

Across all jurisdictions, a small but growing minority (between 7·6 and 27·9 per cent in 2011) of boys had orchidopexy before the recommended age of 1 year, although most underwent the operation by 3 years. There was evidence of socioeconomic inequity in the proportion of boys born after the introduction of guidelines in 2008 who received orchidopexy by 1 year of age, favouring those of higher SEP. There was no clear evidence of inequity for those born before 2008 or for orchidopexy by 3 years of age before or after the introduction of guidelines. The findings showed remarkable consistency across regions.

Each jurisdiction had its own measure of SEP (*Table* *S1*, supporting information)^33^, ^34^. However, relative comparisons between quintiles within countries should be stable, rendering within‐country estimates reliable. There were small numbers of cases within each quintile, hence the necessity to dichotomize SEP, which may have masked more subtle gradations. The administrative data resources do not have adequate data on referrals and so it was not possible to analyse separate parts of the pathway between referral and treatment. It may, for example, be that any observed inequities are due to later diagnosis, referral or decision to treat. Despite possible different referral pathways in each jurisdiction or in the availability of operating theatre space, these results were consistent across jurisdictions, suggesting that results may be similar in other countries.

Undescended testis is a relatively common condition, which was clearly and consistently coded within jurisdictions. It is screened for at birth and that process is not associated with SEP (after excluding preterm births and boys with congenital anomalies). A limitation in the diagnostic system used, however, is that it was not possible to separate congenital from acquired cryptorchidism. In this study, operations beyond 5 years of age were censored to minimize the inclusion of acquired cryptorchidism, but it seems probable that such patients may explain some of the high rates of late operation. This study spanned a sufficiently long period to cover the introduction of international guidelines in 2008 and therefore captured what should have been a wave of innovation adoption across all SEP groups.

Other studies^15^, ^16^, ^17^, ^19^ have noted that guidelines are not being met routinely. In the present study, it was observed not only that this was a persisting issue but that, by using comparable methods across jurisdictions, it was consistent in different countries. There are several potentially overlapping reasons for non‐adherence. Operating after 1 year of age may result from delayed diagnosis^15^ or lack of knowledge about the recommended age of orchidopexy, thus causing later referral and older age at orchidopexy^14^. Healthcare systems must balance the need to perform early orchidopexy against the need to carry out other procedures, where there may also be delays and non‐adherence to timing guidelines^35^. Evidence about long‐term fertility and malignancy risk, particularly regarding whether orchidopexy should be performed at less than 1 year or later, is still uncertain given the limitations of existing studies, with small sample sizes or proxy outcomes. In a systematic review^14^ evaluating the optimal age for orchidopexy, only two^36^, ^37^ of 24 studies examined fertility as opposed to a proxy. As for the association with malignancy, current literature has focused mostly on children undergoing orchidopexy at a later age (comparing children having the procedure at age above 10 or 13 *versus* less than 10 or 13 years)^7^, and it remains unclear whether findings showing that earlier surgery reduces risk for children aged under 10 years can be extrapolated to infants. The present findings of overwhelming non‐adherence might in part be due to these limitations in the evidence, particularly if practitioners are weighing the risks of long‐term harm with the risks and contraindications of early surgery^38^. This should therefore lead to the generation of stronger and more robust evidence of the balance of harms and benefits according to the timing of orchidopexy.

This study also showed socioeconomic inequities, which occurred most clearly after introduction of the guidelines. This suggests possible inequities in the adoption of the ‘innovation’ regarding surgery in the first year. Diffusion of innovation in healthcare takes time^39^, and it may be that those in better socioeconomic circumstances, as a result of either their own resources or interactions with the healthcare system, are more likely to be ‘early adopters’ through better access to the healthcare system or being more informed or questioning parents^29^. It has been suggested^28^, in the context of waiting times, that people with a higher SEP, by having higher levels of education and social capital, may be better equipped to articulate their needs and engage actively in waiting list systems. As these people are likely to have better working conditions, it might also be easier for them to attend appointments^28^. This is not to suggest that people with a lower SEP are less likely to seek healthcare, as there is evidence to the contrary^24^, but that they may be at a disadvantage when dealing with a complex system. If the findings of the present study are the result of diffusion of innovation, it may be that those with a lower SEP will ‘catch up’ and close the currently modest inequities in age at orchidopexy.

Across five jurisdictions, orchidopexy was not performed consistently before a child's first birthday, as recommended by international guidelines, for the majority of patients. Although lack of adherence to recommended practice may reflect balancing the risks of delaying surgery with those of performing an operation under general anaesthesia early in life, there may be systems‐level factors, such as limited availability of hospital space, that cause delay. Evidence of long‐term harms associated with performing orchidopexy before or after 1 year of age, however, remains very weak. Long‐term monitoring of outcomes and further research into the precise timing of events within the diagnosis–referral–treatment pathway still need to be carried out. National and international guidelines should be reassessed to account better for the risks of early *versus* delayed surgery.

## Supporting information


**Appendix** **S1** Congenital anomaly ICD‐10 codes

**Appendix** **S2** Cumulative incidence and proportions of orchidopexy cases

**Appendix** **S3** Characteristics of birth cohorts by region

**Appendix** **S4** Inequity ratios in time to surgery

**Appendix** **S5** Sensitivity analyses for cumulative incidence and proportions of cases

**Appendix** **S6** Sensitivity analyses by region

**Table S1** Description of the data sets
