# Supplementary material for: Timing of paediatric orchidopexy in universal healthcare systems: international administrative data cohort study
Source: BJS Open. 2020 Jul 24;4(6):1117–24. doi: 10.1002/bjs5.50329 (PMC7709362; doi:10.1002/bjs5.50329)
Supplement: Supplementary file 7 — Table S1 Description of the data sets [file BJS5-4-1117-s007.docx]

**BJS5_50329**

**Timing of paediatric orchidopexy in universal healthcare systems: international administrative data cohort study**

**M. A. Jay, A. Arat, L. Wijlaars, O. Ajetunmobi, T. Fitzpatrick, H. Lu, S. Lei, C. Skerritt, S. Goldfeld, M. Gissler, G. Gunnlaugsson, S. Hrafn Jónsson, A. Hjern, A. Guttmann and R. Gilbert**

**Appendix S1** Congenital anomaly ICD-10 codes

**Appendix S2** Characteristics of birth cohorts by region

**Appendix S3** Characteristics of birth cohorts by region

**Appendix S4** Inequity ratios in time to surgery

**Appendix S5** Sensitivity analyses for cumulative incidence and proportion of cases

**Appendix S6** Sensitivity analyses by region

**Table S1** Description of the data sets

| **Country** | **Description** | **Years*** | **Cryptorchidism diagnosis codes** | **Orchidopexy procedure codes** | **Socioeconomic position indicator** |
| --- | --- | --- | --- | --- | --- |
| England | The Hospital Episode Statistics Admitted Patient Care (HES APC) dataset, provided by NHS Digital, was used in England. HES APC covers all inpatient admissions, including maternity & birth admissions and day cases, funded by the English National Health Service (NHS). The birth cohorts for England were constructed using a method previously described.^40^ Diagnosis and procedure coding is performed by trained hospital clinical coders on patient discharge. | 2003-2011 | ICD-10: Q53 (all subcodes), Q55.0 & Q55.1. These codes were used in all regions. | OPCS-4: N08 & N09 (all sub-codes) | English Index of Multiple Deprivation quintiles at birth: an area-based measure of deprivation based on neighbourhoods with an average population of 1,000 to 3,000 people.^41^ |
| Finland | Boys with cryptorchidism were identified from the Register on Congenital Anomalies. Information on premature birth was taken from the Medical Birth Register. Operations for cryptorchidism was identified in the Care Register for Health Care (Hospital Discharge Register), which includes inpatient overnight stays, day care as well as outpatient specialised care. The unique ID number was used to link information between the registers. | 2004-2011 |  | NOMESCO v 1.15: KFH00, KFH10, JAH01 | Maternal occupation at birth:  (1) upper white collar;  (2) lower white collar;  (3) blue collar; (4) other including students and homemakers. |
| Iceland | Data were retrieved from the Birth Registry and Hospital Discharge Registry, provided by the Directorate of Health. It includes all male births in Iceland and all ICD-10 diagnosis of retentio testis with operations performed in the two main hospitals in the country (University Hospital and Akureyri Hospital); no information was available for operations done in private clinics. The data with unique personal ID number were transferred to Statistics Iceland (SI), who linked them with data in Registries Iceland and the tax registry. Subsequently, recoded data without the unique ID number were made accessible by SI in their web-portal Idunn, and analysed in R. | 2003-2011 |  | NOMESCO v 1.15: KFH00, KFH10, JAH01 | Equivalent total family income (quintiles) for male births compared to income of all families in the population. |
| Ontario, Canada | Ontario, Canada’s largest province, is home of ICES, which houses a breadth of linked health administrative and demographic data for all legal Ontario residents. Each resident has a unique coded personal identifier, allowing for linkage and longitudinal follow-up across multiple databases. Birth cohorts were created using the MOMBABY database, a linked database of maternity and newborn records covering all births in the province since 1988. The Canadian Institute for Health Information’s (CIHI) Discharge Abstract Database (DAD) was used to identify hospital diagnosis and procedure codes. See <http://datadictionary.ices.on.ca> for the data dictionary. | 2003-2011 |  | Canadian Classification of Health Interventions (CCI). 1QM74DA, 1QM74LA, 1QM74LAKD | Ontario Marginalization (ON-Marg), Material Deprivation Index quintiles: an area-based measure of marginalisation based on neighbourhoods of 400 to 700 people.^42^ |
| Scotland | Data from Scotland consisted of several linked datasets from the Information Services Division Scotland. These were the National Records for Scotland – Births and Deaths (NRS); the Scottish Morbidity Records – Maternity (SMR02); the Scottish Birth Record (SBR); the Scottish Morbidity Records (SMR11); and the Scottish Morbidity Records for General and Acute Specialties (SMR01). Collectively these are known as the SMR, which covers all Scottish NHS-funded inpatient stays including day cases. A process diagram showing how the birth cohorts for Scotland were constructed can be found in the on-line material. Diagnosis and procedure coding is performed by trained hospital clinical coders on patient discharge. | 2003-2011 |  | OPCS-4: N08 & N09 (all sub-codes) | Scottish Index of Multiple Deprivation quintiles at birth: an area-based measure of deprivation based on neighbourhoods of 500 to 1,000 people.^43^ |
| Sweden | Operation for cryptorchidism was identified in the National Patient Register, which includes inpatient overnight stays, day care as well as outpatient specialised care. Data on household income was obtained from the Swedish Longitudinal Integration Database for Health Insurance and Labour Market studies. The unique ID number was used to link information on parental variables from the Swedish Multi-Generation Register. | 2003-2011 |  | NOMESCO v 1.15: KFH00, KFH10, JAH01 | Disposable household income quintiles, based on all taxed income and transfers minus taxes paid divided by the number of consumer units in the household. This index is created by Statistics Sweden according to an algorithm that includes all incomes in the household reported to Swedish Tax Agency, subtracted by taxes and divided by consumer units. |
| Victoria, Australia | Hospital admission and clinical data were obtained from the Victorian Admitted Episodes Dataset (VAED). The VAED is an administrative dataset containing data on all admitted patient activity submitted by all public and private acute hospitals, including acute facilities in rehabilitation and extended care institutions and day procedure centres. | 2005-2013* |  | ACHI: 37803-00, 37803-01, 37604-04, 37604-05, 37604-07, 37604-08, 37809-00, 37809-01, 30390-00 | N/A |

ISD Information Services Division; ICD-10 International Classification of Diseases and Related Health Problems 10^th^ Revision; NOMESCO Nordic Medico-Statistical Committee Classification of Surgical Procedures; OPCS-4 Office of Population Censuses and Surveys Classification of Interventions and Procedures version 4; ACHI Australian Classification of Health Interventions.

*All years are birth years except Victoria, Australia, where birth cohorts were not available.

**The superscript numbers in the above table refer to references in the reference list of the main article.**
